# Glomus Tumor of the Cheek: A Case Report

**DOI:** 10.1155/2012/307294

**Published:** 2012-07-02

**Authors:** Konstantinos Veros, Konstantinos Markou, Chrysa Filitatzi, Dionysios E. Kyrmizakis

**Affiliations:** ^1^1st Department of Otorhinolaryngology Head & Neck Surgery, AHEPA University Hospital, 54006 Thessaloniki, Greece; ^2^Department of Pathology, General Hospital of Veroia, 59100 Veroia, Greece; ^3^Department of Otorhinolaryngology Head & Neck Surgery, General Hospital of Veroia, 59100 Veroia, Greece

## Abstract

Glomus tumors are benign, subcutaneous neoplasms of the perivasculature. Though facial location is rare, the diagnosis of a glomus tumor should be considered in cases of undiagnosed painful facial nodules or chronic facial pain. Imaging aids in defining the tumor and planning a complete excision in order to avoid recurrence. Histological examination is mandatory after every attempted excision. A case of glomus tumor of the cheek along with the possible pitfalls of diagnosis and treatment and a brief review of the limited associated literature are presented.

## 1. Introduction

Glomus tumors are benign neoplasms of the perivasculature accounting for 1-2% of soft tissue tumors [1]. They usually present as painful subcutaneous nodules commonly described in the subungual area of the digits. Though various other locations have been described, glomus tumors of the cheek are rare. In a reported study of 56 extradigital glomus tumors seen in Mayo Clinic over a period of twenty years (1985–2005), the authors found a single case occurring in the cheek [2]. Apart from the classic triad of symptoms described for glomus tumors, pain, localised tenderness, and cold hypersensitivity, the rare occurrence of glomus tumor in the cheek can be the cause of undiagnosed chronic facial pain. We present a case of a glomus tumor of the cheek mentioning the issues we encountered and briefly review the limited available literature for this rare entity.

## 2. Case Report

**Figure 1:**
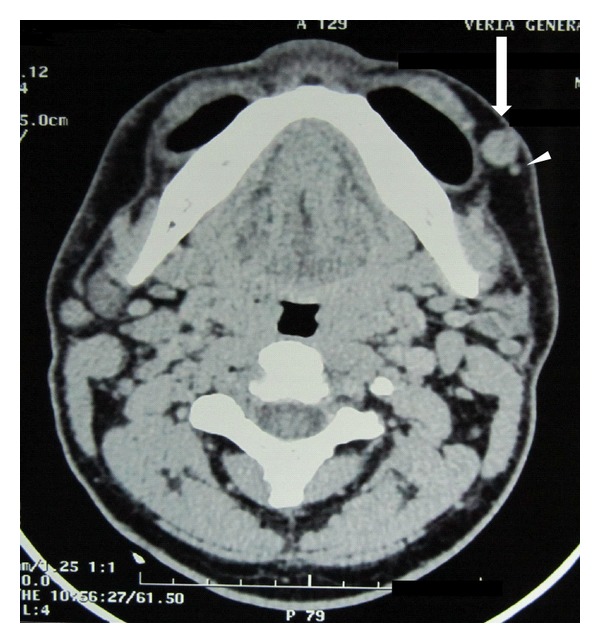
Contrast-enhanced CT (puffed cheek method) revealed a well-defined, enhanced, round soft-tissue mass of the left cheek over the buccinator muscle (arrow) measuring 13.8 × 9 mm along with a tiny satellite lesion (arrowhead).

**Figure 2:**
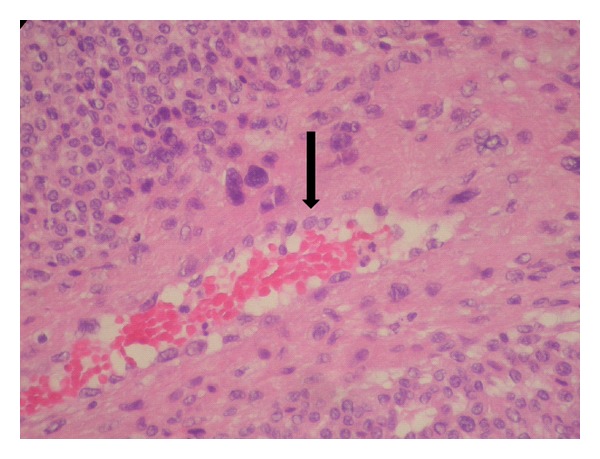
Hematoxylin and eosin stain, ×40. Glomus tumor of the solid type. Infiltration of the vessel wall by the neoplastic cells (arrow) does not indicate malignancy.

**Figure 3:**
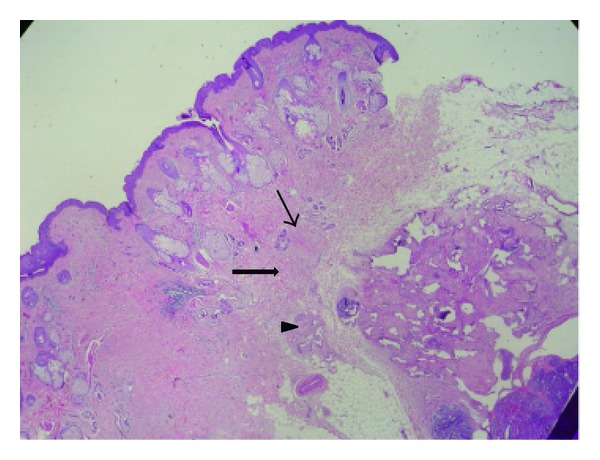
Hematoxylin and eosin stain, ×10. Glomus tumor with solid (thin arrow), angiomatoid (arrowhead), and angiomyomatous (arrow) features.

A 24-year-old man presented at our clinic with a four-year history of a painful nodule in his left cheek that reoccurred after two attempted excisions in the last four months by different specialists. No biopsy result or other histological information was available. Otherwise healthy, the patient complained of pain and local tenderness. Clinical examination revealed a well-defined, round, firm nodule in the middle of the left cheek, approximately 1 × 1 cm in size that appeared to be subdermic with no apparent fixation to the underlying tissues. The overlying skin was normal and the nodule was extremely painful on palpation. Examination of the oral cavity was normal and no neck lymph nodes could be palpated. Complete head and neck examination and laboratory tests were also unremarkable. Contrast enhanced CT (puffed cheek method) revealed a well-defined, contrast-enhanced, round soft-tissue mass of the left cheek over the buccinator muscle measuring 13.8 × 9 mm, with no apparent relations to the surrounding tissues (Figure 1). Attached to it a very small satellite lesion was noted. In the operating room under local anesthesia, a relatively wide excision in order to remove both the masses was performed. Though we were not able to demarcate the main and satellite nodules macroscopically, no evidence of residual disease was apparent. Special care was taken regarding the protection of facial nerve branches and the cosmetic result. Histological examination of the specimen was consistent with the diagnosis of glomus tumor of the solid type, with no mention of potential residual disease (Figure 2). The patient reported relief of his symptoms after surgery, but at 2-month followup he complained of gradual recurrence of local tenderness at the site of the excision. Three months later, the patient had a small palpable nodule and complete recurrence of his symptoms. Under local anesthesia, a very wide excision including the previous scar (with approximately 1 cm margin around the scar) was performed. Histological examination revealed glomus tumor with solid, angiomatoid, and angiomyomatous features (Figure 3). The margins of the excision were reported as free of lesion. The patient remains without any symptoms nine months after surgery.

## 3. Discussion

Glomus tumors are neoplasms of the glomus body, a neuromyoarterial unit found within the reticular dermis that serves as a specialized arteriovenous anastomosis [3]. The arterial end of the glomus body or Sucquet-Hoyer canal is surrounded by modified smooth muscle cells called glomus cells that act to regulate blood flow to the skin in response to temperature changes [3–5]. The normal glomus body was first described by Hoyer in 1877, whereas Masson [6] in 1924 provided the first clinical description of a glomus tumor. Histologically, depending on the predominant component, there are three variants of glomus tumor, namely: (1) solid, with poor vasculature and scant smooth muscle component; (2) angiomatoid (glomangioma), with a predominant vascular component; (3) glomangiomyoma, with prominent vascular and smooth muscle components [7].

Accounting for 1-2% of soft tissue tumors [1], glomus tumors present mostly as solitary masses with a rarer multiple variant [8, 9]. Malignant transformation is extremely rare. Glomus bodies are most highly concentrated in the digits, palms, and soles of the feet [4], and glomus tumors are most commonly described in the upper extremity and especially in the subungual area of the digits. While extradigital glomus tumors are not a rare subgroup of glomus tumors [2] and various locations have been described, facial location and especially in the cheek is rare and only a few cases have been reported in the literature [2, 10–12].

The symptoms and signs of a facial glomus tumor may vary from an asymptomatic (rarely) subcutaneous nodule to excruciating chronic facial pain. The most expected presentation is that of a small, painful, subcutaneous nodule. A long history of consultations by various specialists without a definite diagnosis can be expected. The classic triad of symptoms described for glomus tumors consisting of pain, localised tenderness, and cold hypersensitivity may or may not be present. Overlying skin may or may not be discoloured. Though usually not larger than 1 × 1 cm in size, large facial glomangiomas mimicking venous malformations have been reported [10]. A 4 : 1 male predominance has been reported for extradigital glomus tumors [2].

Magnetic resonance imaging has proven to be the most sensitive imaging modality for the diagnosis of glomus tumors in the extremities [13–15]. Contrast enhanced CT can aid in the differential diagnosis and in delineating the anatomic relations necessary for operative management. Because of their small size and subcutaneous location, glomus tumors are particularly amenable to complete removal thus making surgical excision the treatment of choice. Histological examination is necessary to confirm the diagnosis, and documentation should be provided to the patient. Though excellent results can be anticipated, recurrence rates vary from 12% to 33% [16–19]. As in our case, it is not clear whether recurrence represents inadequate excision or the presence of multiple tumors not detected at initial assessment of the patient. Recurrence within days to weeks of surgery may suggest inadequate excision [18]; in contrast, symptoms 2 to 3 years postoperatively may indicate multiple tumors [13, 17]. On account of the limited associated literature, further data regarding specifically facial glomus tumors are needed.
